# Identification of Novel Flavonoids and Ansa-Macrolides with Activities against *Leishmania donovani* through Natural Product Library Screening

**DOI:** 10.3390/pathogens13030213

**Published:** 2024-02-28

**Authors:** Trong-Nhat Phan, Hyeryon Lee, Kyung-Hwa Baek, Joo Hwan No

**Affiliations:** 1 Institute of Applied Science and Technology, School of Technology, Van Lang University, Ho Chi Minh City 700000, Vietnam; nhat.pt@vlu.edu.vn; 2 Faculty of Applied Technology, School of Technology, Van Lang University, Ho Chi Minh City 700000, Vietnam; 3 Host-Parasite Research Laboratory, Discovery Biology, Institut Pasteur Korea, Seongnam-si 13488, Gyeonggi-do, Republic of Korea; hyeryon.lee@ip-korea.org (H.L.); kyunghwa.baek@ip-korea.org (K.-H.B.)

**Keywords:** *Leishmania*, natural product, high-throughput screening, flavonoid, ansa-macrolide

## Abstract

The protozoan parasite *Leishmania donovani* is the causative agent of visceral leishmaniasis (VL), a potentially fatal disease if left untreated. Given the limitations of current therapies, there is an urgent need for new, safe, and effective drugs. To discover novel antileishmanial compounds from previously unexplored chemical spaces, we conducted a high-throughput screening (HTS) of 2562 natural compounds, assessing their activity against *L. donovani* promastigotes and intracellular amastigotes. Utilizing the criteria of ≥70% parasite growth inhibition and ≥70% host cell (THP-1) viability, we selected 100 inhibitors for half-maximal inhibitory concentration (IC_50_) value determination. Twenty-six compounds showed activities in both forms of *Leishmania* with a selectivity index of over 3. Clustering analysis resulted in four chemical clusters with scaffolds of lycorine (cluster 1), 5-hydroxy-9,10-dihydro-4*H*,8*H*-pyrano[2,3-*f*]chromene-4,8-dione (cluster 2), and semi-synthetic derivatives of ansamycin macrolide (cluster 4). The enantiomer of lycorine, BMD-NP-00820, showed the highest anti-amastigote activity with an IC_50_ value of 1.74 ± 0.27 μM and a selectivity index (SI) > 29. In cluster 3, the most potent compound had an IC_50_ value of 2.20 ± 0.29 μM with an SI > 23, whereas in cluster 4, with compounds structurally similar to the tuberculosis drug rifapentine, BMD-NP-02085 had an IC_50_ value of 1.76 ± 0.28 μM, but the SI value was 7.5. Taken together, the natural products identified from this study are a potential source for the discovery of antileishmanial chemotypes for further development.

## 1. Introduction

Protozoan parasites of the genus *Leishmania* are etiological agents for leishmaniasis, with transmission facilitated by *Phlebotomus* and *Lutzomyia* sandflies in the Old and New Worlds, respectively [1,2]. The life cycle of *Leishmania* is characterized by two principal forms: promastigotes, which inhabit in the alimentary canal of the sandfly vector, and intracellular amastigotes, which reside within the macrophages of the mammalian host [3]. Leishmaniasis maintains endemic status across over 97 countries globally, presenting in three primary clinical manifestations: cutaneous leishmaniasis (CL), mucocutaneous leishmaniasis, and visceral leishmaniasis (VL). The most severe form, VL, predominates in regions such as Ethiopia, South Sudan, India, and Brazil, with an estimated 400,000 novel cases each year [4,5,6,7].

The current therapeutic regimens for leishmaniasis, including antimonials, miltefosine, amphotericin B, and paromomycin, are constrained by route of administration, drug resistance, cost-prohibitive factors, or associated toxicities. For instance, pentavalent antimonials (SbV) such as sodium stibogluconate (Pentostam^®^) and meglumine antimoniate (Glucantime^®^) are still the first choice of drug to treat patients with VL in some countries, but the drug, which is delivered through the intravenous or intramuscular routes, can lead to serious, sometimes life-threatening, adverse effects such as cardiotoxicity, pancreatitis, and nephrotoxicity [8]. Moreover, sodium stibogluconate showed a failure rate from 59% to 65% in the treatment of VL patients in Bihar, India, due to drug resistance rendering it ineffective in curing the disease [9,10,11]. As exemplified by the case of SbV, there is an exigent need for the development of novel, safe, and efficacious treatments for leishmaniasis [12,13,14].

Natural products have historically constituted a foundational element in the pharmacopeia for antiparasitic and antibacterial therapeutics, evidencing their indispensable role in addressing infectious diseases. Quinine, extracted from the bark of *Cinchona* spp., which was recently elucidated to target the purine nucleoside phosphorylase of the *Plasmodium* parasite, served as a cornerstone in the treatment of malaria, demonstrating the profound impact of phytochemicals in antiparasitic interventions [15]. Artemisinin, a sesquiterpene lactone extracted from *Artemisia annua*, further exemplifies the critical contribution of natural products, offering potent antimalarial efficacy [16]. In the case of leishmaniasis, amphotericin B, a polyene antibiotic produced by *Streptomyces nodosus* that has been initially approved for use against fungal infections, is now instrumental in treating leishmaniasis through a liposomal formulation. Paromomycin, an aminoglycoside isolated from *Streptomyces krestomuceticus* used in clinics to treat leishmaniasis, amebiasis, giardiasis, and tapeworm infection, exhibits potent antileishmanial activity through the inhibition of protein synthesis by binding to the parasite’s ribosomal RNA [17]. Ivermectin, a macrocyclic lactone derived from *Streptomyces avermitilis*, represents a breakthrough in the management of onchocerciasis and lymphatic filariasis, attributing to its potent anthelmintic activity [18]. Additionally, rifampicin, a bactericidal antibiotic produced by *Streptomyces mediterranei*, has been integral in multidrug regimens for tuberculosis, highlighting the versatility of natural products in combating bacterial pathogens [19]. Even with the mentioned notable examples, natural products are still a source of highly underexplored chemical space that can serve as templates for novel antileishmanial pharmacophores.

Recognizing the significance of natural products in antiparasitic research, numerous studies have sought to harness natural product extracts for their activity against *Leishmania* parasites. A recent investigation has revealed that apigenin (4′,5,7-trihydroxyflavone), a flavonoid ubiquitously present in various fruits and vegetables, exhibits potent activity against intracellular amastigotes with an inhibitory concentration (IC_50_) of 2.3 µM and a selectivity index of 34.3 [20]. This compound has also been demonstrated to effectuate a 99.7% reduction in hepatic parasites in a murine model of visceral leishmaniasis [20]. Additionally, a natural product extract identified as dodeca-2*E*,4*E*-dienoic acid 4-hydroxy-2-phenylethylamide, isolated from the roots of *Anacyclus pyrethrum*, has been reported to exhibit antileishmanial activity against *L. donovani* amastigotes as well as *Trypanosoma brucei* and *T. cruzi*, with IC_50_ values of 13.3, 7.17, and 5.97 µM, respectively [21]. In the work of Mishra et al., a compound termed prenyloxy-naphthoquinone, extracted from the roots of *Plumbago zeylanica*, displayed EC_50_ values against both the promastigote and amastigote forms of *L. donovani* that were significantly lower than those of the standard drug miltefosine [22]. Another compound, methyl-ent-3β-hydroxylabd-8(17)-en-15-oate, derived from *Piliostigma thonningii*, demonstrated inhibitory activity against *L. donovani* amastigotes and *T. brucei*, with IC_50_ values of 7.82 and 3.84 µM, respectively [23].

The exemplified cases stem from the conventional approach of assay-guided fractionation followed by structural determination. However, such methods can often lead to a loss of activity during fractionation or to the re-identification of previously reported compounds posing difficulties in the discovery process. To bypass these hurdles, in this study, we utilized a unique and diverse natural product library composed of 2562 compounds, designed using cheminformatics, to directly assess antileishmanial activity and identify inhibitors, rather than identifying a single active agent through assay-guided fractionation processes. The in vitro high-throughput screening (HTS) of this library against *L. donovani* promastigotes and intracellular amastigotes, followed by structural analysis, has identified novel chemical entities with antileishmanial activities.

## 2. Materials and Methods

### 2.1. Maintenance of Parasites and Mammalian Cells Lines

*L. donovani MHOM/SD/62/1S-CL2D* parasites were cultured as promastigotes at 28 °C in M199 medium (Sigma-Aldrich) supplemented with 40 mM HEPES, 0.1 mM adenine, 0.0001% biotin, and 4.62 mM NaHCO_3_, supplemented with 10% fetal bovine serum (FBS), 100 U/mL penicillin, and 100 µg/mL streptomycin. Human acute monocytic leukemia (THP-1) cells (American Type Culture Collection, THP-1 TIB-202) were cultured in RPMI-1640 medium containing 4.5 g/L glucose, 10 mM HEPES, 1 mM sodium pyruvate, and 10% FBS. These cells were maintained in a 5% CO_2_ incubator at 37 °C [24]. Both parasites and mammalian cell lines were sub-cultured every 2 or 3 days and maintained for no more than ten passages.

### 2.2. Chemicals and Inhibitors

Amphotericin B and miltefosine were purchase from Sigma-Aldrich (Saint Louis, MO, USA). The natural product compounds were all purchased from Pharmeks Ltd. (Moscow, Russia).

### 2.3. Screening of Natural Compounds against L. donovani Promastigote Viability Assay

The inhibition of *L. donovani* promastigote growth was assessed by measuring the conversion of resazurin to resorufin. The promastigotes were dispensed at a density of 5 × 10^4^ *L. donovani* promastigotes per well into 384-well plates and exposed to the natural product library composed of 2562 molecules at 10 μM for 72 h. Following the incubation, resazurin sodium salt was added, and the plates were further incubated for 5 h before fixation using 4% paraformaldehyde. The results were then analyzed using a Victor3™ plate reader (PerkinElmer, Shelton, CT, USA) at an emission wavelength of 590 nm and an excitation wavelength of 530 nm. Amphotericin B and miltefosine served as reference drugs for inhibiting the growth of *L. donovani* promastigotes. The hits identified from the screening were confirmed by testing in a 10-point, 1/2 dilution manner starting from 400, 200, 100, or 50 μM (depending on the solubility and activity) in duplicates of two independent experiments to calculate the IC_50_ values.

### 2.4. Screening of Natural Compounds against L. donovani Intracellular Amastigote

PMA-treated THP-1 cells were seeded at 0.8 × 10^4^ cells per well in a 384-well plate in RPMI-1640 complete medium supplemented with 10% FBS. After 48 h of incubation at 37 °C in the presence of 5% CO_2_, the *L. donovani* amastigotes, isolated from infected hamster liver, were added to the cell culture at a ratio of 10:1. The infected THP-1 cells were treated with screening compounds (the same set used in the promastigotes HTS) at 10 µM, with amphotericin B and miltefosine serving as positive controls and 0.5% DMSO as a negative control. After 72 h, the cells and parasites were washed, stained, and fixed with 5 µM DAPI and 4% PFA. Images were acquired through Operetta^®^ CLS^™^ (PerkinElmer, Shelton, CT, USA), and the numbers of parasites, host cells, and infection ratios were quantified using the Columbus^™^ software (https://www.perkinelmer.com/product/image-data-storage-and-analysis-system-columbus, accessed on 7 February 2024) (PerkinElmer, Shelton, CT, USA). The hits identified from the screening were confirmed by testing in a 10-point, 1/2 dilution manner starting from 400, 200, 100, or 50 μM in duplicates of two independent experiments to calculate the IC_50_ values.

### 2.5. Ethic Statement

All animal studies were performed in strict accordance with the guidelines and principles established by the Korean Animal Protection Law (https://elaw.klri.re.kr/, accessed on 8 Feburary 2024). The use of hamster for isolating *Leishmania* amastigotes for the in vitro infection was approved by the Institutional Animal Care and Use Committee (IACUC) of the Institut Pasteur Korea (IACUC approval number IPK-16003).

### 2.6. Statistical Analyses

All of the IC_50_ and the half-maximal cytotoxic concentration (CC_50_) values were calculated using two independent experiments. Outliers were excluded, and the dose–response curves were fitted using the GraphPad Prism V6.0 software (GraphPad Software, San Diego, CA, USA) by using a sigmoidal dose–response equation with a variable hill slope option.

## 3. Results

### 3.1. Screening of the Natural Product Library against L. donovani Promastigotes and Amastigotes

**Figure 1:**
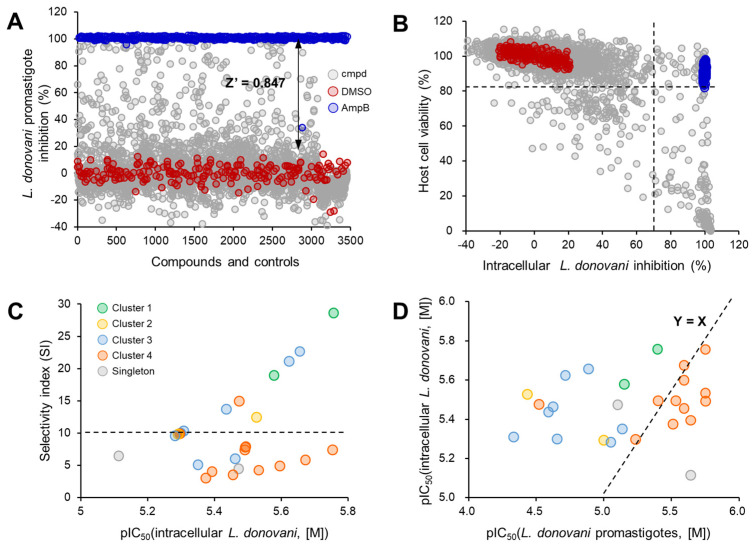
High−throughput screening (HTS) of a natural-product-based library against *L. donovani* and profiling of activities by clusters. (**A**) HTS of a 2562-natural-compound library against *L. donovani* promastigotes with Z′ factor. (**B**) Anti−amastigote versus host cell (THP−1) viability bi−plot of HTS, resulting in the intracellular *Leishmania* model. (**C**) Plotting of anti-amastigote activities (pIC_50_) with the selectivity index. (**D**) Plotting of pIC_50_ values of compounds from intracellular *L. donovani* versus *L. donovani* promastigote assays.

A library composed of 2562 natural compounds selected from the Dictionary of Natural Products through unsupervised clustering to ensure diversity was screened at 10 μM against *L. donovani* promastigotes. The quality of the screening was assessed by calculating the Z′ prime value as follows: Z′ = 1 − 3(σ_c_^+^ + σ_c_^−^)/(μ_c_^+^ − μ_c_^−^), where σ_c_^+^/σ_c_^−^ were the standard deviation values of the positive/negative controls, and μ_c_^+^/μ_c_^−^ were the corresponding mean values. The Z′ prime value of 0.847 implied that the HTS assay was excellent (Figure 1A). Eighty-six compounds were active based on a ≥70% inhibition, with a hit rate of 3.36%. The Z′ value of HTS for *L. donovani* intracellular amastigotes was 0.747 (Figure S1). Based on a ≥70% threshold of inhibition of intracellular parasite survival and a ≥70% host cell viability, 41 compounds were selected out of 2562, with an overall hit rate of 1.6% (Figure 1B). We then applied the criteria of ≥70% inhibition in either extracellular or intracellular *L. donovani* growth and ≥70% host cell viability to select 100 hits for IC_50_ determination, of which 59 were promastigote-specific, 14 were amastigote-specific, and 27 were active in both forms.

### 3.2. Clustering of Compounds and Profiling of Antileishmanial Activities

**Figure 2:**
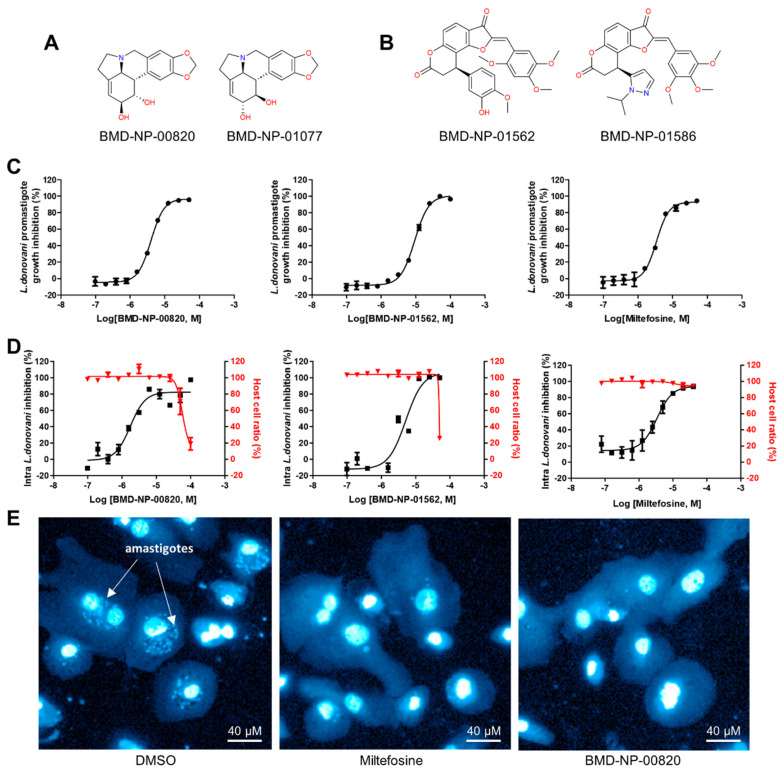
Chemical structure of compounds from clusters 1 and 2 and in vitro antileishmanial activity. (**A**) Chemical structure of lycorine and its enantiomer, cluster 1. (**B**) Chemical structure of cluster 2. (**C**) Dose–response curve (DRC) of BMD−NP−00820, BMD−NP−01562, and miltefosine in the promastigote assay and in the intracellular *Leishmania* assay (**D**). (**E**) Representative fluorescence image of DAPI−stained intracellular amastigotes treated with DMSO, miltefosine, or BMD−NP−00820.

**Figure 3:**
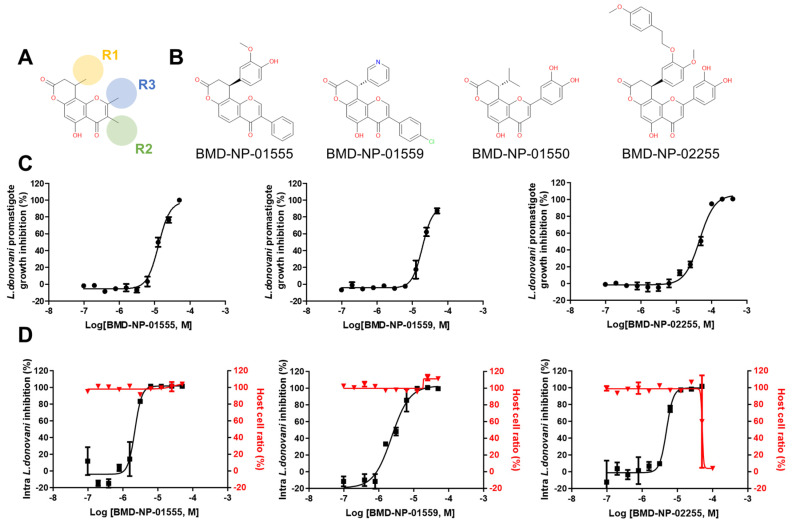
Chemical structure of compounds from cluster 3 and in vitro antileishmanial activity. (**A**) The structure of the chemical scaffold, 5−hydroxy−9,10−dihydro−4*H*,8*H*−pyrano[2,3−*f*]chromene−4,8−dione, of cluster 3. (**B**) The structure of representative compounds from cluster 3. (**C**) Dose–response curve (DRC) of BMD−NP−01555, BMD−NP−01558, and BMD−NP−01559 in the promastigote assay and in the intracellular *Leishmania* assay (**D**).

**Figure 4:**
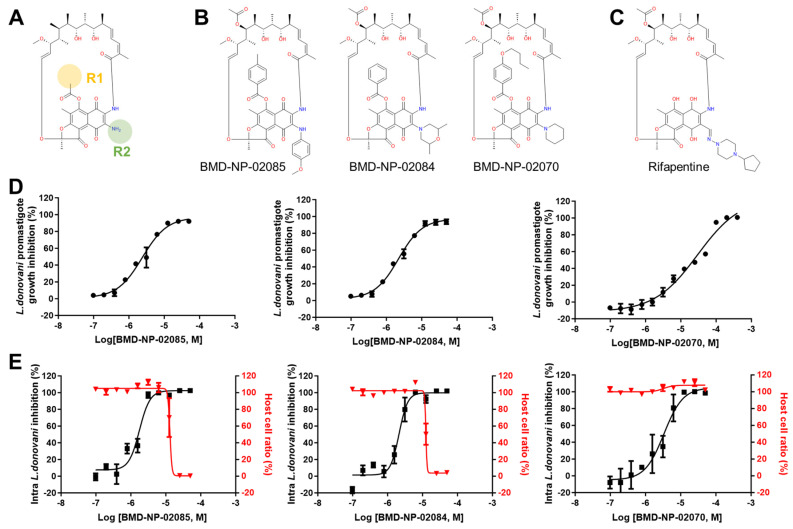
Chemical structure of compounds from cluster 4, ansa−macrolides, and in vitro antileishmanial activity. (**A**) The structure of the chemical scaffold of identified ansa−macrolides. (**B**) The structure of representative compounds from cluster 4. (**C**) The chemical structure of rifampentine. (**D**) DRC of BMD−NP−02085, BMD−NP−02084, and BMD−NP−02070 in the promastigote assay and in the intracellular *Leishmania* assay (**E**).

Among the confirmed compounds, we further selected 26 compounds that were confirmed active in both promastigotes and amastigotes with a selectivity index (SI; THP-1 half-maximal cytotoxic concentration (CC_50_)/amastigotes IC_50_) > 3 (Table 1). Chemical structure clustering yielded four clusters and two2 singletons. Cluster 1 included lycorine and its enantiomer, while the scaffold of cluster 2 was identified as 8,9-dihydro-7*H*-furo[2,3-*f*]chromene-3,7(2*H*)-dione. Cluster 3 was characterized by a 5-hydroxy-9,10-dihydro-4*H*,8*H*-pyrano[2,3-*f*]chromene-4,8-dione scaffold, and cluster 4 comprised semi-synthetic derivatives of ansamycin macrolide (Figure 2A,B, Figure 3A and Figure 4A). The compounds in clusters 1 and 3 showed high SIs (>10), but the values were relatively low for cluster 4 (Figure 1C). The -OH enantiomer of lycorine, BMD-NP-00820, was the most potent compound, with an IC_50_ value of 1.74 ± 0.27 μM against amastigotes, having the highest SI > 29. Lycorine, BMD-NP-01077, was slightly less active, with an IC_50_ value of 2.63 ± 0.57 μM, but it still had a high SI of over 19 (Figure 1C and Figure 2D,E). In terms of activity in promastigotes versus amastigotes, the compounds in clusters 1 and 3 were mostly more active in the promastigote form, up to 12 times in terms of IC_50_ values, but for the compounds in cluster 4, the activities were similar between the forms as the points were located near the Y = X line (Figure 1D).

### 3.3. Activity and Structural Analysis of Flavonoids (Cluster 3)

Based on the structural clustering, eight flavonoids were included in cluster 3. The scaffold was identified as 5-hydroxy-9,10-dihydro-4*H*,8*H*-pyrano[2,3-*f*]chromene-4,8-dione with substitutions at the R^1^, R^2^, and R^3^ positions (Figure 3A). The IC_50_ range was 2.20~5.21 μM for amastigotes and 7.24~45.9 μM for promastigotes. The most active compound, BMD-NP-01555, showed an IC_50_ of 2.20 ± 0.29 μM and a CC_50_ > 50 μM in the amastigote assay and 12.86 ± 0.28 μM in the promastigote assay. This compound had a methoxyphenol at the R^1^ position and a benzene ring at the R^2^ position (Figure 3B). The compound, BMD-NP-01550, with pyrocatechol at the R^3^ position and isobutane at the R^1^ position, showed toxicity with a CC_50_ of 20.84 ± 1.04 μM. In general, the compounds in this cluster showed relatively potent activities against amastigotes as well as a low in vitro toxicity.

### 3.4. Activity and Structural Analysis of Ansa-Macrolides (Cluster 4)

In cluster 4, there were 12 ansa-macrolides that were structurally similar to the anti-tubercular drug rifampentine (Figure 4B,C). Substitutions at the R^1^ and R^2^ positions were observed, where the most potent compound, BMD-NP-02085, with an IC_50_ of 1.76 ± 0.28 μM, had methylbenzene at the R^1^ position and methoxybenzene at the R^2^ position (Figure 4B,E). This compound was relatively cytotoxic, with a CC_50_ of 13.07 μM (SI = 7.4). Interestingly, the compounds with butoxybenzene at the R^1^ position, BMD-NP-02070 and -02096, showed a low toxicity, with a CC_50_ of over 50 μM (Figure 4B,E). The compounds in cluster 4 displayed similar activities against promastigotes and amastigotes, but their toxicity against the host THP-1 cells varied depending on the substitutions.

## 4. Discussion

Natural products serve as an attractive source of structurally diverse compounds for drug discovery against *Leishmania* parasites [25,26,27,28]. For instance, amphotericin B, originally used for fungal infections, has been repurposed for leishmaniasis. This compound is known to bind to ergosterol, a sterol not present in humans, to form pores that lead to the killing of parasites and fungi. With the liposomal formulation, AmBisome^®^, the side effects were significantly reduced, and a single-dose cure was achieved for leishmaniasis patients [29]. Paromomycin, an aminoglycoside used for bacterial infections, is another example of a natural-product-based leishmaniasis drug now approved for use in India [30]. It is known to target the *Leishmania* ribosome, and recently, the structural elucidation of the inhibitor–ribosome complex has opened new potential for the further optimization of the drugs and the development of novel chemical entities [31]. Among the four leishmaniasis drugs, these two natural-product-based drugs play a pivotal role in treating patients along with miltefosine and antimonials, the latter of which is becoming ineffective due to drug resistance.

In recent years, there has been significant progress in VL drug discovery, where compounds identified through a phenotypic approach have been further optimized for clinical development. An extensive mode of action investigation has led to the identification of drug targets. For example, GSK3186899/DDD853651, based on the pyrazolopyrimidine scaffold, was found to be active against *Leishmania*. Through chemical proteomics and genomics approaches, cyclin-dependent kinase 12 was elucidated as the target of the compound [32]. GSK3494245/DDD01305143 and LXE408, which are in clinical development, were found to selectively target the proteasome β5 subunit of kinetoplastid parasites, whereas the human ortholog was not inhibited by the compound [33,34,35]. DNDi-6148, discovered through screening of the oxaborole class of compounds, is another example. Mode-of-action studies have confirmed that the polyadenylation specificity factor 3 of *Leishmania* is the target of the compound [36]. These examples highlight the critical role of discovering new chemical entities, which can pave the way for uncovering innovative drug targets for advanced development. With the importance of identifying new chemical entities, natural products possessing antileishmanial properties present a unique avenue, as they occupy a distant chemical space when compared to synthetic small molecules. This distinction is readily observed in the structures of amphotericin B and paromomycin, which differ from those of other synthetic antileishmanial compounds. And this characteristic has the potential to reveal targets associated with unique mechanisms of action that can be utilized for further discoveries. Thus, the continuous discovery of novel antileishmanial natural products expects to provide templates for the identification of new drug targets for leishmaniasis.

In the field of natural product drug discovery, the traditional method involves bioassay-guided fractionation starting from crude extracts [37]. However, this approach can lead to a loss of activity during fractionation, the re-identification of previously reported compounds, and an insufficient supply of starting biological material [38,39]. To bypass these hurdles and efficiently identify natural products with antileishmanial activity, we utilized a library of natural products selected from the Dictionary of Natural Products through unsupervised clustering to cover diverse chemotypes. The screening of this library, composed of 2562 single components against *L. donovani* promastigotes and intracellular amastigotes, resulted in the identification of 26 compounds belonging to four clusters and two singletons.

Cluster 1 was composed of two compounds, lycorine and its enantiomer. Both compounds exhibited IC_50_ values in the low single-digit µM range with no cytotoxicity measured in THP-1 cells up to 50 µM. Recently, an extract from a plant collected in Peru, *Clinanthus milagroanthus*, was found to be active against the promastigote form of *L. braziliensis*, with an IC_50_ of 3.5 µg/mL [40]. This extract was identified to have lycorine as a major component; however, this single component was neither tested against the parasite nor against its amastigote form. Further, the extract was tested in an in vivo model of cutaneous leishmaniasis using hamsters and showed a 90% decrease in lesion size [40]. Based on our findings, lycorine is expected to be the active agent exerting antileishmanial activity both in vitro and in vivo.

Cluster 3 comprised compounds with the 5-hydroxy-9,10-dihydro-4*H*,8*H*-pyrano[2,3-*f*]chromene-4,8-dione scaffold, including eight compounds that were structurally similar to the group of compounds known as calanolides. Calanolides E1 and E2, derived from the stem barks of *Calophyllum brasiliense*, were reported to display activity against *L. infantum* with IC_50_ values of 37.1 and 29.1 µM, respectively, and E2 was shown to have a low toxicity with an SI > 6.9 [41]. The compounds we identified, for example, BMD-NP-01555, showed exceedingly high potency with an IC_50_ = 2.2 µM and a higher SI of over 23. Given that this class of compounds generally showed a low toxicity with a high activity, and due to the low complexity of their structure, further chemical optimization could be possible to generate inhibitors potentially applicable in in vivo studies.

The compounds in cluster 4 were ansa-macrolides that were structurally similar to the anti-tubercular class of rifamycins (rifampetine, rifampicin, rifampin, and rifabutin), initially identified from *Amycolaptopsis rifamycinica* (Figure S2). Rifamycin selectively targets prokaryotic RNA polymerase, with the binding constant being 100 times higher (indicating a lower affinity) for eukaryotic RNA polymerase [42]. There have been studies regarding the antileishmanial efficacy and activity of rifampicin in humans, and experimental and in vitro models; however, the results were rather contradictory, with variations in species [43,44,45,46]. In this study, the ansa-macrolides identified were structurally very similar to rifamycins, with substitutions in the R^1^ and R^2^ groups. As different chemical moieties at the R^1^ and R^2^ positions only lead to minor differences in antileishmanial activity, these two classes of molecules may share the same mode of action.

In conclusion, the screening of a natural product library composed of single components in this study has efficiently identified new chemotypes with antileishmanial activities and low cytotoxicity. A brief structure–activity relationship was investigated with flavonoid and ansa-macrolide scaffolds. These findings, including the scaffolds, can be further utilized to develop more effective and safe inhibitors against *Leishmania* through the help of medicinal chemistry and could be applied to other kinetoplastid parasites.

## Figures and Tables

**Table 1:** In vitro activity of identified inhibitors from the promastigote and intracellular *Leishmania* assays.

No.	Compounds	*L. donovani* Promastigote Inhibition IC_50_ (µM)	Intracellular *L. donovani* Inhibition IC_50_ (µM)	THP-1 Cell Ratio CC_50_ (µM)	Selectivity Index (SI)	Cluster #
1	BMD-NP-00820	3.99 ± 0.40	1.74 ± 0.27	>50.00	>28.7	1
2	BMD-NP-01077	6.99 ± 0.74	2.63 ± 0.57	>50.00	>19.0	1
3	BMD-NP-01586	36.34 ± 0.42	2.97 ± 0.33	36.90 ± 1.13	12.4	2
4	BMD-NP-01562	9.88 ± 0.71	5.10 ± 0.34	>50.00	>9.8	2
5	BMD-NP-01555	12.86 ± 0.28	2.20 ± 0.29	>50.00	>22.7	3
6	BMD-NP-01559	19.08 ± 0.13	2.37 ± 0.49	> 50.00	>21.1	3
7	BMD-NP-01550	23.54 ± 0.52	3.44 ± 0.64	20.84 ± 1.04	6.1	3
8	BMD-NP-02287	25.51 ± 0.23	3.65 ± 0.89	>50.00	>13.7	3
9	BMD-NP-01581	7.24 ± 0.33	4.45 ± 0.62	22.88 ± 0.92	5.1	3
10	BMD-NP-02255	45.9 ± 1.09	4.90 ± 0.65	>50.00	10.4	3
11	BMD-NP-02249	21.85 ± 0.57	5.01 ± 0.71	48.10 ± 2.06	10.0	3
12	BMD-NP-01584	8.83 ± 0.99	5.21 ± 0.28	>50.00	>9.6	3
13	BMD-NP-02085	1.76 ± 0.28	1.76 ± 0.85	13.07 ± 0.56	7.4	4
14	BMD-NP-02084	2.53 ± 0.14	2.12 ± 0.40	12.45 ± 1.02	5.9	4
15	BMD-NP-02086	2.53 ± 0.11	2.53 ± 0.52	12.50 ± 0.76	4.9	4
16	BMD-NP-02101	1.76 ± 0.14	2.93 ± 0.57	12.45 ± 0.91	4.3	4
17	BMD-NP-02097	3.93 ± 0.42	3.20 ± 0.27	25.38 ± 1.23	7.9	4
18	BMD-NP-02094	2.9 ± 0.35	3.20 ± 0.27	25.01 ± 1.31	7.8	4
19	BMD-NP-02095	1.75 ± 0.44	3.22 ± 0.69	24.02 ± 1.24	7.5	4
20	BMD-NP-02070	29.93 ±1.27	3.34 ± 0.42	>50.00	>15.0	4
21	BMD-NP-02100	2.53 ± 0.51	3.49 ± 0.66	12.52 ± 0.71	3.6	4
22	BMD-NP-02069	2.25 ± 0.20	4.03 ± 0.14	16.45 ± 1.16	4.1	4
23	BMD-NP-02068	3.04 ±0.40	4.21 ± 0.28	12.77 ± 0.83	3.0	4
24	BMD-NP-02096	5.75 ± 0.86	5.03 ± 0.42	>50.00	>9.9	4
25	BMD-NP-00940	7.84 ± 0.85	3.36 ± 0.49	15.13 ± 1.01	4.5	s
26	BMD-NP-02265	2.26 ± 0.14	7.67 ± 0.93	>50.00	>6.5	s

## Data Availability

Data are contained within the article and Supplementary Materials.

## Supplementary Materials

The following supporting information can be downloaded at: https://www.mdpi.com/article/10.3390/pathogens13030213/s1, Supplementary Information Figure S1: HTS results of a 2562-natural-compound library against intracellular *L. donovani* with Z′ factor; Supplementary Information Figure S2: Structure of compounds identified from this study and anti-tubercular drug rifamycins.
