# Supplementary figures and images for: Identification of Novel Flavonoids and Ansa-Macrolides with Activities against Leishmania donovani through Natural Product Library Screening
Source: Pathogens. 2024 Feb 28;13(3):213. doi: 10.3390/pathogens13030213 (PMC10974828; doi:10.3390/pathogens13030213)

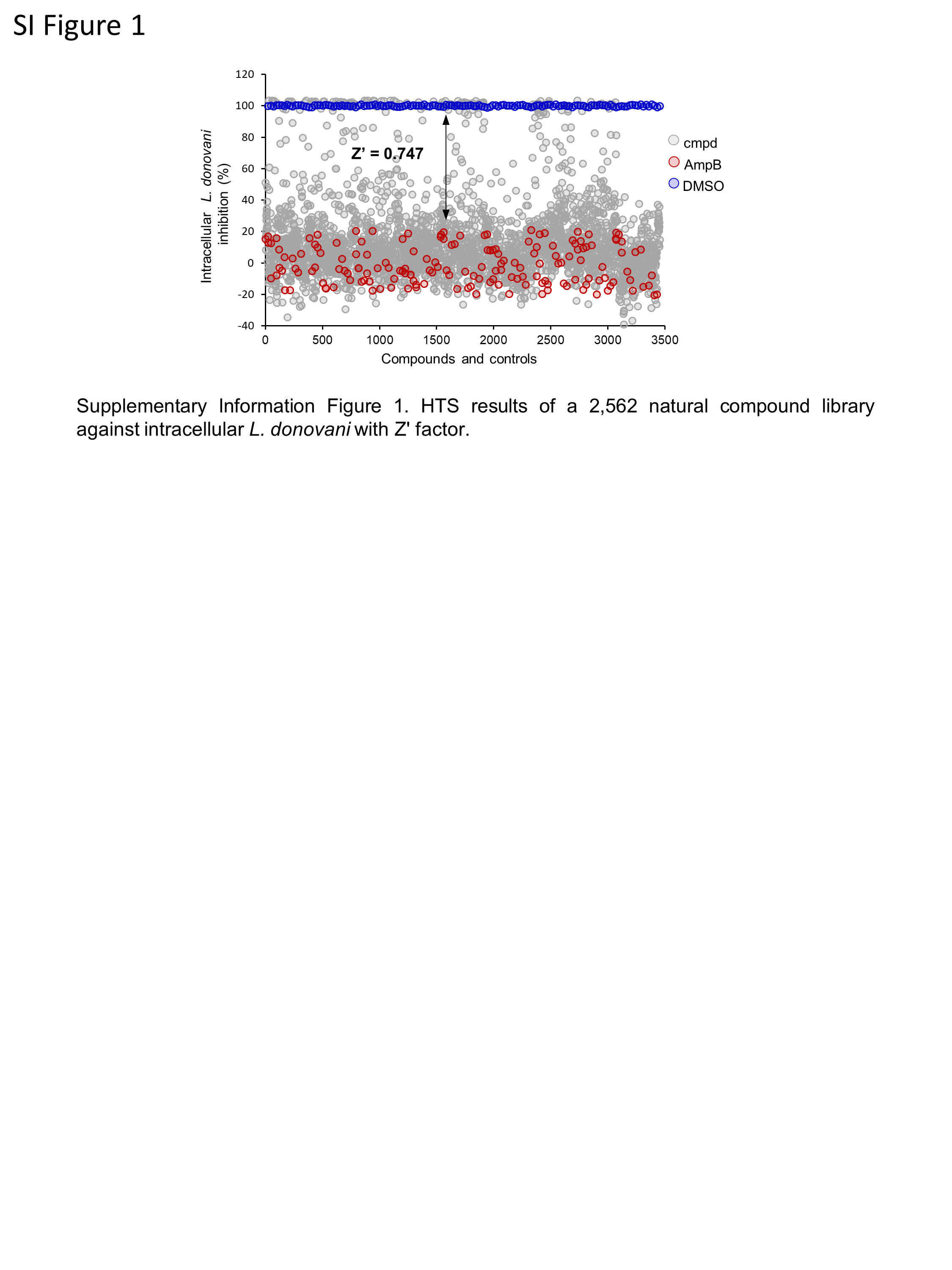

Supplement: Supplementary file 1 [file pathogens-13-00213-s001.zip › LeishNP_SI_figure1.TIF]

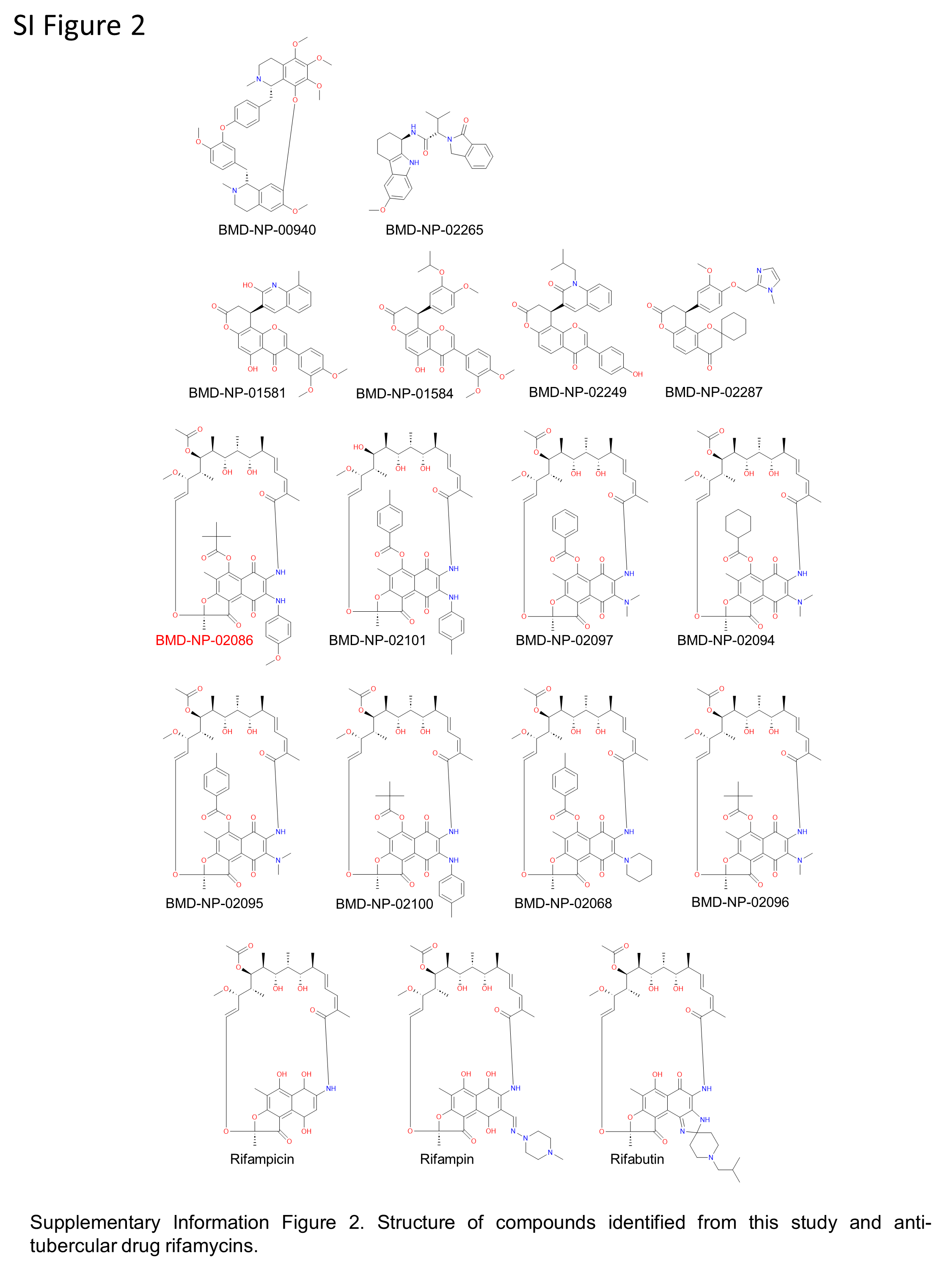

Supplement: Supplementary file 1 [file pathogens-13-00213-s001.zip › LeishNP_SI_figure2.TIF]
